# Variability of adenoidectomy/tonsillectomy rates among children of the Veneto Region, Italy

**DOI:** 10.1186/1472-6963-9-25

**Published:** 2009-02-07

**Authors:** Ugo Fedeli, Maria Marchesan, Francesco Avossa, Francesco Zambon, Marilisa Andretta, Iacopo Baussano, Paolo Spolaore

**Affiliations:** 1 SER-Epidemiological Department, Veneto Region, Castelfranco Veneto (TV), Italy; 2 ORL Unit, Azienda ULSS 8, Asolo (TV), Italy; 3 Department of Infectious Disease Epidemiology, St. Mary's Campus, Imperial College London, London, UK; 4 CPO-Piemonte, Novara, Piemonte, Italy

## Abstract

**Background:**

Despite national guidelines in 2003 aimed at limiting the recourse to tonsillectomy and/or adenoidectomy (A/T), the latter are among the most frequent pediatric surgeries performed in Italy. Aim of the study is to investigate variability of A/T rates among children of the Veneto Region, Italy.

**Methods:**

All discharges of Veneto residents with Diagnosis-Related Groups 57–60 and ICD9-CM intervention codes 28.2 (tonsillectomy), 28.3 (adenotonsillectomy), 28.6 (adenoidectomy) were selected in the period 2000–2006 for a descriptive analysis. A multilevel Poisson regression model was applied to estimate Incidence Rate Ratios (IRR) with 95% Confidence Intervals (CI) for A/T surgery among children aged 2–9 years in 2004–2006, while taking into account clustering of interventions within the 21 Local Health Units.

**Results:**

Through 2000–2006, the overall number of A/T surgeries decreased (-8%); there was a decline of adenoidectomies (-20%) and tonsillectomies (-8%), whereas adenotonsillectomies raised (+18%). Analyses on children aged 2–9 resulted in an overall rate of 14.4 surgeries per 1000 person-years (16.1 among males and 12.5 among females), with a wide heterogeneity across Local Health Units (range 8.1–27.6). At random intercept Poisson regression, while adjusting for sex and age, intervention rates were markedly lower among foreign than among Italian children (IRR = 0.57, CI 0.53–0.61). A/T rates in the 10–40 age group (mainly tonsillectomies) computed for each Local Health Unit and introduced in the regression model accounted for 40% of the variance at Local Health Unit level of pediatric rates (mainly adenoidectomies and adenotonsillectomies).

**Conclusion:**

A/T rates in the Veneto Region, especially adenoidectomies among children aged 2–9 years, remain high notwithstanding a decrease through 2000–2006. A wide heterogeneity according to nationality and Local Health Units is evident. The propensity to A/T surgery of each Local Health Unit is similar in different age groups and for different surgical indications.

## Background

Tonsillectomies and/or adenoidectomies (A/T) are among the most frequent pediatric surgeries performed in Italy [1]; in 2001 they accounted for 28% of surgical hospitalizations in children aged 0–14 [2]. National guidelines issued in 2003 restricted the surgical option mainly to: children with significant obstructive apnea (adenotonsillectomy), children with recurrent otitis media and ventilation-tube placement or with chronic/recurrent sinusitis and failure of appropriate antibiotic therapy (adenoidectomy), children and adults with severe acute recurrent tonsillitis (tonsillectomy) [1].

The first claim of high tonsillectomy rates and geographical variability unexplained by medical indications was issued in England and Wales as early as in 1938 [3]. 70 years later, wide variation between and within countries continues to be registered [4]. The adoption of guidelines with clear indications for surgery should overcome such differences in surgical rates, at least in an area with mild heterogeneity in the delivery of health care such as the Veneto Region, Italy. Aim of the study is to investigate variability of A/T rates among children of the Veneto Region.

## Methods

The Veneto Region has about 4700000 inhabitants and is subdivided in 21 Local Health Units. Each child can have access to a family pediatrician who provides ambulatory care and refers to other specialists in case of need; hospital care is covered by general taxation and extends to all residents (irrespective of citizenship). In each Local Health Unit at least one acute care hospital is equipped with an otorhinolaryngological unit performing A/T surgeries (Table 1). Data on A/T surgeries were retrieved from the 2000–2006 regional archive of hospital discharge records; we extracted all discharges of residents in Veneto with Diagnosis-Related Groups 57–60 (tonsillectomy and adenoidectomy procedures) and ICD9-CM intervention codes 28.2 (tonsillectomy), 28.3 (adenotonsillectomy), 28.6 (adenoidectomy). The distribution of the three surgical procedures by sex and age was graphically illustrated in order to identify the age range where intervention numbers were higher.

**Table 1:** Population, otorhinolaryngological units and hospital beds, number of tonsillectomies (T), adenotonsillectomies (T+A), adenoidectomies (A) performed on children aged 2–9, years 2004–2006.

	Min	Max	Median
	
Total population	74908	453216	206002
Population 2–9 yrs	4292	33935	14726
% non-Italians, age class 2–9 yrs	4%	16%	9%
n. otorhinolaryngological units	1	3	1
Total hospital beds available in otorhinolaryngological units	10	44	21
T (2–9 yrs)	10	97	35
T+A (2–9 yrs)	116	684	254
A (2–9 yrs)	65	872	389

Median and range across Local Health Units of the Veneto Region.

Data on the mid-year population by sex, age, Local Health Unit and citizenship were computed from data provided by the National Institute of Statistics [5]. Since the pediatric population is rapidly changing in our Region mainly due to immigration, and data from the National Institute of Statistics on non-national population is available only for more recent years, further analyses were restricted to children aged 2–9 operated in 2004–2006. Crude rates of A/T surgery as a whole, adenoidectomy, tonsillectomy with or without adenoidectomy were computed for each Local Health Unit. A random-intercept Poisson regression model was applied to estimate Incidence Rate Ratios (IRR) with 95% confidence Intervals (CI) for A/T surgery associated with age, sex, and citizenship (Italian and non-Italian), while taking into account clustering of interventions within Local Health Units. Finally, a cluster-level variable (overall A/T rates of each Local Health Unit in the 10–40 year age class) was introduced in the hierarchical model to assess if it could account for part of the variability between Local Health Units in pediatric A/T rates.

## Results

**Figure 1:**
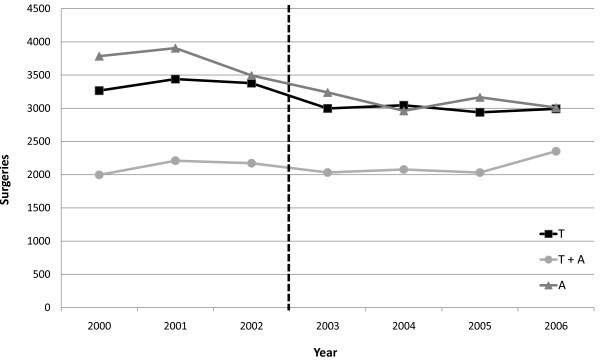
**Number of tonsillectomies (T), adenotonsillectomies (T+A), and adenoidectomies (A) performed in the Veneto Region, 2000–2006**. National guidelines introduced in 2003 (dashed line).

**Figure 2:**
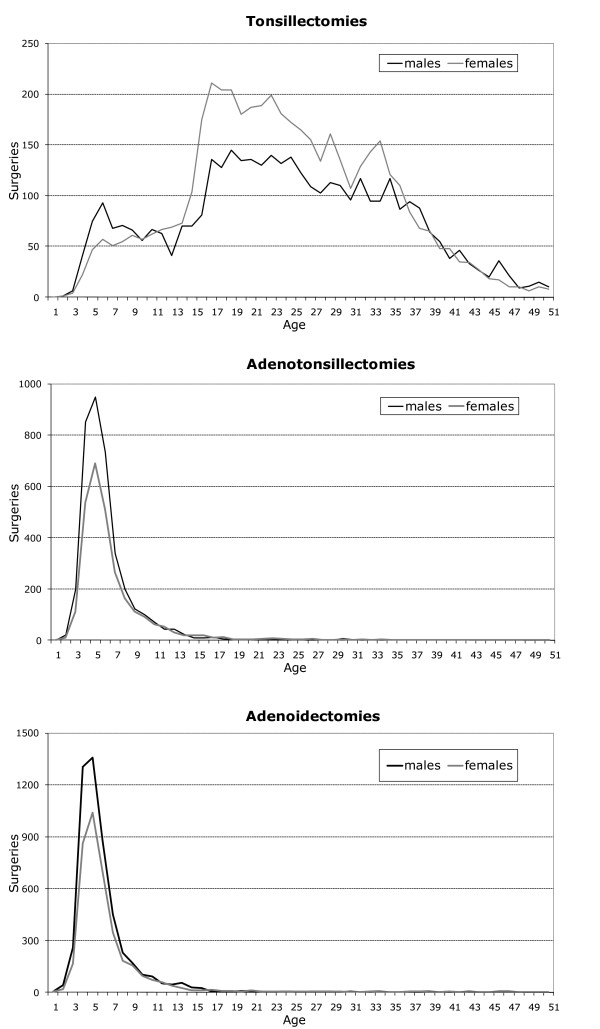
**Age and sex distribution of surgeries, Veneto residents, 2004–2006**.

Through 2000–2006, the overall number of A/T surgeries performed in the Veneto Region declined until 2003, thereafter remaining stable (Figure 1); the overall decrease of A/T surgeries (-8%) was accounted by adenoidectomies (-20%) and tonsillectomies (-8%), whereas adenotonsillectomies raised (+18%). Figure 2 shows the number of surgeries performed on residents in Veneto by sex and age in 2004–2006. Adenotonsillectomies and adenoidectomies were limited to children aged less than 10 years (peaking at 4 years), mainly males. Tonsillectomies showed a minor peak in males < 10 years, thereafter steeply increased in adolescent and young adults, especially females.

**Figure 3:**
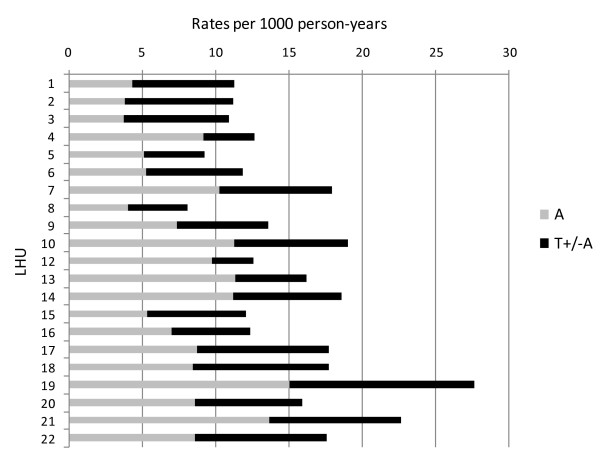
**Children aged 2–9 years, Veneto Region, 2004–2006: rates of adenoidectomy (A) and tonsillectomy or adenotonsillectomy (T+/-A) by Local Health Unit (LHU)**.

Analysis on children aged 2–9 in 2004–2006 included 15096 surgeries (830 tonsillectomies, 5974 adenotonsillectomies, 8292 adenoidectomies); the above age range accounted for 92% of adenotonsillectomies and adenoidectomies, but only for 9% of tonsillectomies performed on residents in Veneto in the study period. The corresponding regional A/T rate was 14.4 per 1000 person-years (16.1 among males and 12.5 among females). Heterogeneity across Local Health Units was wide (Figure 3), with rates ranging from 8.1 to 27.6 per 1000 person-years; such heterogeneity included both adenoidectomies (regional rate 7.9 per 1000 person-years, range 3.8–15.1) and tonsillectomies with or without adenoidectomy (regional rate 6.5 per 1000 person-years, range 2.9–12.5). 9.6% of children living in Veneto were non-nationals, with a corresponding overall A/T rate of 8.8 per 1000 person-years (14.9 among Italians). At random intercept Poisson regression (Table 2), while adjusting for sex and age, A/T intervention rates among non-Italians reached only 57% of the value observed among Italian children (IRR = 0.57, CI 0.53–0.61). If A/T rates in the 10–40 age group (mainly tonsillectomies) of each Local Health Unit were introduced in the regression model, such variable was statistically significant (p < 0.001), and the variance at Local Health Unit level in pediatric rates (mainly adenoidectomies and adenotonsillectomies) decreased by 40% (from 0.082 to 0.048). The ratio between hospital beds available in otorhinolaryngological units and total (or pediatric) population was not associated to Local Health Unit surgical rates (data not shown). It is worth of notice that the difference in intervention rates among non-national children was greater for adenoidectomies (IRR = 0.47, CI 0.43–0.52) than for tonsillectomies with or without adenoidectomy (IRR = 0.70, CI 0.64–0.76).

**Table 2:** Age-adjusted Incidence Rate Ratios (IRR) with 95% Confidence Intervals (CI) for tonsillectomy and/or adenoidectomy (A/T) among children aged 2–9 years.

	IRR	(95% CI)
	
Males vs females	1.29	(1.25–1.33)
Non-Italian vs Italian children	0.57	(0.53–0.61)
A/T rates (×1000) in each LHU, age class 10–40 yrs	1.51	(1.22–1.86)

LHU-level variance = 0.048.

Random-intercept Poisson regression; clustering variable = Local Health Unit (LHU).

## Discussion

The regional archive of hospital discharge records includes hospitalizations in both public and private hospitals, as well as hospitalizations of Veneto residents in facilities outside the region. Since A/T surgeries are performed in hospital (mainly as day-case, or with a single overnight stay) and not in outpatient settings in Italy, the hospital discharge records archive allows a complete analysis of these interventions.

The age and sex distribution of A/T surgeries found in the Veneto region is similar to that reported from other European countries [6,7]; the observation of higher intervention rates in male children is consistent since the first paper issued in 1938 [3].

Usually age and sex standardized intervention rates are reported separately for tonsillectomy (with or without adenoidectomy) and for adenoidectomy. We maintained the above distinction; however since there are two distinct peaks, around age 4 for adenotonsillectomy and adenoidectomy and among adolescents-young adults for tonsillectomy, we preferred to restrict the age range of most analysis and deal with a homogenous population in terms of type of surgery and demographics (e.g. relevance of the immigrant population).

A/T rates show a wide variability between countries [8,9], but it has been long recognized that, despite differences in average rates across countries, even more striking variations always exist from area to area of the same country [10-13]. A wide small-area variation in intervention rates suggests that there is a high degree of "clinical uncertainty" about the indications [14]. In Italy, high heterogeneity in tonsillectomy rates between regions, only in part related to minimum temperatures registered in regional capitals, was observed in 2000 [15]. After the introduction of national guidelines for A/T surgery in 2003, a reduction in the number of interventions and in the inter-regional variability of rates has been registered [16]. However, data from the Veneto Region suggest that the decline in the number A/T surgeries (mainly adenoidectomies) started before guidelines were issued, and disappeared in recent years. Moreover, wide small-area variation in intervention rates still persists. In our analyses we did not take into account factors that may be related to surgery rates at the Local Health Unit level, such as accessibility of primary health care or resource available in otorhinolaryngological units. The hierarchical model applied to our data shows a direct relationship between A/T rates in children aged 2–9 years and in adolescent-young adults aged 10–40 years, even though main surgical indications and type of operation are different between these age groups. This finding suggests that variability between Local Health Units is better explained by surgeons' general propensity to tonsillectomy and adenoidectomy procedures rather than by different underlying disease rates. All the above indicate that national guidelines did not have a major impact on the selection of children for surgery, similarly to that observed in other countries [13]. An update of national guidelines has been issued in 2008, further restricting indications to ventilation-tube placement and adenoidectomy in recurrent otitis [16], but strategies should be implemented for enhancing adherence among physicians, who could see guidelines both as a threat to their decisional autonomy [17] and as a cost-cutting tool for health care. Few surgeons are hesitant believers in the necessity and efficacy of the operations they perform, yet feedback of population-based surgical rates showing how much such certainties vary from place to place [18] should convince of the need to reevaluate patterns of patient selection at the local level [19].

The association between socio-economic status and A/T rates has been investigated mostly at an ecological level, with some studies showing higher rates related to a lower socioeconomic level [20], including one investigation carried out in 1997 in Rome, Italy, claiming that disadvantaged groups are more vulnerable in receiving unnecessary surgical treatments [21]. The hospital discharge records archive did not permit to include socioeconomic indicators in our analysis. However, the relation between socioeconomic status and intervention rates may change over time and space; the steep increase of the immigrant population in Italy in the last few years (about threefold from 1997 to 2006) could partially overcome earlier observations. In our study A/T rates are strikingly lower in the non-national (or foreign) population, which consists of persons who still have the nationality of their home country and may include persons born in Italy. Separate analyses for foreign children born within or outside the Veneto Region could have been of great interest but were not feasible due to the lack of denominator data (population by citizenship: children born in Italy from foreign parents maintain their parents' citizenship). Nonetheless, the proportion of foreign children born in the Veneto Region is rapidly growing (about 18% of newborns in Veneto are currently non-Italians), and -except for housing conditions-shares most environmental risk factors (climate, air pollution, schooling) with the Italian children. Still, being born in a foreign country or having foreign parents can be considered as a proxy variable for the socioeconomic condition. Differences in A/T rates between Italian and non-national residents could be due to different rates of the underlying diseases, but could be better explained by different patterns of diagnosis and care. The latter hypothesis is reinforced by investigations on other types of discretionary surgeries in Italy (e.g. elective cesarean section), showing lower rates among the foreign population [22]. Lower tonsillectomy rates in children from neighborhoods with larger immigrant population has been also observed in Ontario, Canada, with differences possibly arising at the level of the primary care physician, the specialist, or parental attitudes [23].

## Conclusion

Despite the introduction of national guidelines, a wide heterogeneity in A/T rates according to nationality and Local Health Unit is evident in the Veneto Region. The propensity to A/T surgery of each Local Health Unit is similar in different age groups and for different surgical indications. These findings uncover issues both of variability in medical practices and equity in the access to health care.

## Competing interests

The authors declare that they have no competing interests.

## Authors' contributions

UF conceived the study, participated in its design, drafted the manuscript, MM collected the data and performed the statistical analysis, FA planned, performed and revised the statistical analysis, FZ participated in the interpretation of data and drafting of the manuscript, MA conceived the study and participated in its design, IB participated in the interpretation of data and drafting of the manuscript, PS participated in the study design and revised the manuscript. All authors read and approved the final manuscript.

## Pre-publication history

The pre-publication history for this paper can be accessed here:


